# Inhaled hydrogen sulfide protects against lipopolysaccharide-induced acute lung injury in mice

**DOI:** 10.1186/2045-9912-2-26

**Published:** 2012-10-01

**Authors:** Simone Faller, Kornelia K Zimmermann, Karl M Strosing, Helen Engelstaedter, Hartmut Buerkle, René Schmidt, Sashko G Spassov, Alexander Hoetzel

**Affiliations:** 1 Department of Anesthesiology and Critical Care Medicine, University Medical Center Freiburg, Hugstetter Str. 55, D-79106, Freiburg, Germany

**Keywords:** Acute lung injury, Hydrogen sulfide, Sepsis, Lipopolysaccharide, Inflammation

## Abstract

**Background:**

Local pulmonary and systemic infections can lead to acute lung injury (ALI). The resulting lung damage can evoke lung failure and multiple organ dysfunction associated with increased mortality. Hydrogen sulfide (H_2_S) appears to represent a new therapeutic approach to ALI. The gas has been shown to mediate potent anti-inflammatory and organ protective effects *in vivo*. This study was designed to define its potentially protective role in sepsis-induced lung injury.

**Methods:**

C57BL/6 N mice received lipopolysaccharide (LPS) intranasally in the absence or presence of 80 parts per million H_2_S. After 6 h, acute lung injury was determined by comparative histology. Bronchoalveolar lavage (BAL) fluid was analyzed for total protein content and differential cell counting. BAL and serum were further analyzed for interleukin-1β, macrophage inflammatory protein-2, and/or myeloperoxidase glycoprotein levels by enzyme-linked immunosorbent assays. Differences between groups were analyzed by one way analysis of variance.

**Results:**

Histological analysis revealed that LPS instillation led to increased alveolar wall thickening, cellular infiltration, and to an elevated ALI score. In the presence of H_2_S these changes were not observed despite LPS treatment. Moreover, neutrophil influx, and pro-inflammatory cytokine release were enhanced in BAL fluid of LPS-treated mice, but comparable to control levels in H_2_S treated mice. In addition, myeloperoxidase levels were increased in serum after LPS challenge and this was prevented by H_2_S inhalation.

**Conclusion:**

Inhalation of hydrogen sulfide protects against LPS-induced acute lung injury by attenuating pro-inflammatory responses.

## Background

Acute lung injury (ALI), and the acute respiratory distress syndrome (ARDS), are two major challenges in clinical practice and both are responsible for high rates of morbidity and mortality amongst intensive care patients [1-3]. A variety of stimuli can initiate ALI, such as mechanical ventilation, hyperoxia, ischemia/reperfusion, transfusion, or polytrauma [3]. Sepsis reflects one of the most important causes of ALI [4]. The underlying strong inflammatory response in sepsis-induced ALI is characterized by the transmigration of immune-competent cells (mostly neutrophils) into the lung interstitium and the alveolar space, and the release of numerous pro-inflammatory cytokines, *e.g.*, interleukin-1β (IL-1β) and macrophage inflammatory protein-2 (MIP-2). As a consequence of the inflammatory process, alveolar structures change, endothelial and alveolar permeability increase and alveolar fluid clearance decreases, thus critically impairing lung function [5]. Currently available treatment options have failed to significantly decrease sepsis related mortality. Therefore, alternative strategies are urgently needed to improve supportive care.

In this respect, hydrogen sulfide (H_2_S) has come to be a focus of interest. H_2_S belongs to the group of gaseous transmitters, along with nitric oxide and carbon monoxide. It is present in the blood and organs of humans and mammals in low micromolar extracellular concentrations. Endogenous H_2_S has been shown to be involved in a series of physiological processes, *e.g.*, inflammation, vasodilatation, neuromodulation, pain perception, as well as in organ protective pathways (reviewed in [6]). Moreover, exogenous application of gaseous H_2_S and H_2_S donors in different animal disease models like ventilator-induced lung injury [7], ischemia/reperfusion injury [8], or oleic acid-induced ALI [9,10], succeeded in exerting organ-protective effects. The underlying mechanism may be explained by the ability of H_2_S to inhibit the activation and transmigration of neutrophil cells and to attenuate the release of pro-inflammatory cytokines.

The aims of the present study were to mimic sepsis-induced ALI by intranasal administration of the endotoxin lipopolysaccharide (LPS) and to examine the role of continuously inhaled H_2_S in this injury model.

## Methods

### Animals and experimental setting

All animal experiments were performed in accordance with the guidelines of the local animal care commission (Ethics Committee University of Freiburg, Freiburg, Germany, permission No. G-07/25) and in conformance with the journals’ requirements for human and animal trials. C57BL/6 N mice weighing 22.4 g (± 0.3 g) were randomly assigned into four experimental groups: (1) intranasal (i.n.) application of endotoxin-free phosphate buffered saline (70 μl) + spontaneous breathing of room air (PBS + air), (2) PBS treatment + spontaneous breathing of air supplemented with 80 parts per million (ppm) hydrogen sulfide (H_2_S, Air Liquide, Kornwestheim, Germany) (PBS + H_2_S), (3) i.n. application of lipopolysaccharide (70 μl dissolved in PBS, LPS; 0,25 ng E.coli 055:B5; Sigma-Aldrich Chemie GmbH, Munich, Germany) + spontaneous breathing of air (LPS + air), (4) LPS treatment + spontaneous inhalation of 80 ppm H_2_S (LPS + H_2_S). Spontaneous breathing of the respective gas mixture started 1 h prior to PBS or LPS instillation. Instillation of PBS or LPS was conducted under short isoflurane anesthesia. Afterwards, mice were again placed in a sealed plexiglass chamber and subjected to either breathe room air or H_2_S for another 6 h. H_2_S concentration was continuously monitored using a portable gas monitor (MX6 iBrid, Industrial Scientific Corporation, Oakdale, PA).

### Tissue sampling and bronchoalveolar lavage

At the end of each experiment all mice were sacrificed by an intraperitoneal, overdosed injection of ketamine (180 mg/kg) and acepromazine (1.8 mg/kg) and additional bleeding. Bronchoalveolar lavage fluid, lung tissue samples, and blood samples were gained and analyzed as described recently [11].

### Cytokine measurements

BAL aliquots were analyzed using interleukin-1β (IL-1β) and macrophage inflammatory protein-2 (MIP-2) ELISA kits (R&D Systems GmbH, Wiesbaden, Germany) according to the manufacturers’ instructions. Serum samples were tested using ELISA kits for myeloperoxidase (MPO) glycoprotein (HK210 ELISA, Hycult biotech GmbH, Beutelsbach, Germany) and IL-1β according to the manufacturers’ instructions.

### Histological examination

The left lung was prepared, conserved, and cut into 12 μm thick cryosections for hematoxylin and eosin (H + E) staining as previously described [11]. From each lung, four representative photos were taken (magnification x200). Five high power fields (HPF) were randomly assigned to each photo. Subsequently, alveolar wall thickness and cellular infiltration were analyzed by Axiovision software (AxioVS40LE, Zeiss, Jena, Germany). For each HPF, the degree of lung damage was determined using a modified ALI score as described earlier [11]: In brief, (a) thickness of the alveolar walls, (b) infiltration or aggregation of inflammatory cells, and (c) hemorrhage were rated in a blinded fashion. Each item was graded according to the following point scale: 0: minimal damage, 1: mild damage, 2: moderate damage, 3: severe damage, 4: maximal damage. The degree of ALI was assessed by the sum of scores for each HPF, ranging from 0 to 12. The average of the sum of each field score per lung was compared among groups.

### Statistical analysis

Experiments were performed with 6–8 mice per group. Group size was defined on the basis of expected neutrophil cell numbers in BAL fluid by power calculations prior to the study. Graphs represent means ± standard error of means (SEM). Data were further analyzed for normal variation prior to one way analysis of variance (ANOVA), followed by the Student-Newman-Keuls posthoc test. In cases of a failed normality test, Kruskal-Wallis ANOVA on Ranks was performed followed by Dunn`s posthoc test. *P* < 0.05 was considered significant.

## Results and discussion

In this study, we clearly demonstrate, that inhalation of H_2_S in low dose prevents the development of acute lung injury. Furthermore, we show that H_2_S treatment substantially reduces local as well as systemic inflammation.

### Hydrogen sulfide prevents lung damage in LPS-induced ALI

**Figure 1:**
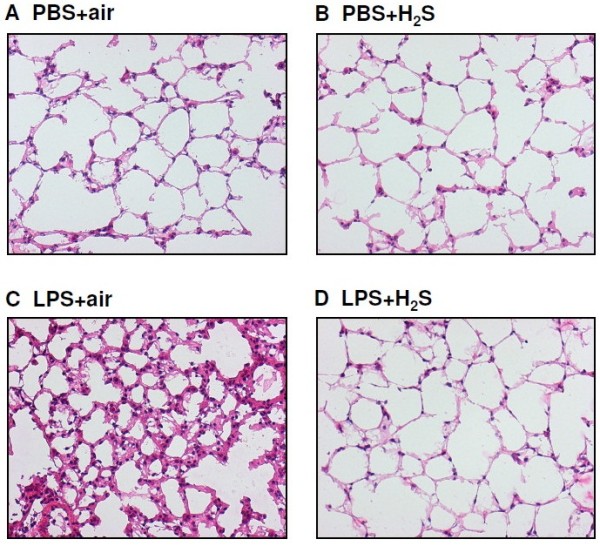
**Effect of LPS and hydrogen sulfide inhalation on lung architecture.** As controls, mice received phosphate buffered saline (PBS, intranasally) and were kept in room air (PBS + air, **A**) or in 80 ppm H_2_S (PBS + H_2_S, **B**) for 6 h (plus 1 h pretreatment). LPS-treated mice (LPS treatment, i.n.) were either kept in room air (LPS + air, **C**) or in 80 ppm H_2_S (LPS + H_2_S, **D**) for 6 h (plus 1 h pretreatment). Sections from the left lung lobe were stained with hematoxylin and eosin. Representative pictures are shown for each experimental group (magnification = 200X).

**Figure 2:**
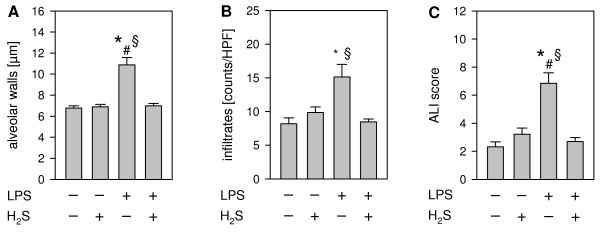
**Effect of LPS and hydrogen sulfide inhalation on lung damage.** As controls, mice received phosphate buffered saline (PBS, intranasally) and were kept in room air or in 80 ppm H_2_S for 6 h (plus 1 h pretreatment). LPS-treated mice (LPS treatment, i.n.) were either kept in room air or in 80 ppm H_2_S for 6 h (plus 1 h pretreatment). Sections from the left lung lobe were stained with hematoxylin and eosin. High power fields were randomly assigned to measure alveolar wall thickness (**A**), to count total infiltrate numbers (**B**), and to calculate an acute lung injury (ALI) score (**C**). Data represent means ± SEM for *n* = 7-8/group. ANOVA on Ranks (**A** + **B**, Dunn`s posthoc test) and ANOVA (**C**; Student-Newman-Keuls posthoc test), *P < 0.05 *vs*. PBS + air group; ^#^*P* < 0.05 *vs*. PBS + H_2_S group; ^§^*P* < 0.05 *vs*. LPS + H_2_S group.

In the presented model of pulmonary sepsis H + E staining of lung cryosections showed that as compared to control conditions (PBS + air, PBS + H_2_S, Figure 1A + B, respectively), LPS treatment clearly stimulated the formation of lung edema and the influx of immune-competent cells (LPS + air, Figure 1C). In sharp contrast, exposure to 80 ppm H_2_S markedly reduced LPS-induced lung damage (LPS + H_2_S, Figure 1D), reflected by decreased edema formation and cellular infiltration into the lung tissue. These findings were confirmed by quantitative analysis: H_2_S inhalation prevented alveolar wall thickening (Figure 2A) and cellular infiltration (Figure 2B), that was otherwise observed in LPS-treated animals kept in room air. In addition, a reduction of lung damage to control levels by H_2_S inhalation was also detected by rating an overall ALI score (Figure 2C), strongly suggesting a lung-protective role for H_2_S inhalation in LPS-induced ALI.

These results add important information to the role of exogenous H_2_S in sepsis. Conflicting data exist in models of cecal ligation and puncture demonstrating aggravation [12-20] as well as substantial reduction of the resulting lung injury in response to application of H_2_S donors [21,22]. It is likely that the route of administration, dosage, timing, and the purity of H_2_S donors may be accountable for the inconsistent data. This study is the first to show that inhalation of H_2_S clearly prevents lung damage due to local LPS-induced injury, underlining the therapeutic potential of this gas under septic conditions. Our findings are further supported by a recent publication by Tokuda et al. [23]. Here, in contrast to our study, LPS was applied systemically. The study found inhalation of 80 ppm H_2_S substantially increased survival. Although the authors did not analyze histopathological changes in the lung, the data suggest an organ-protective effect of H_2_S inhalation. Moreover, we have shown recently that inhalation of H_2_S in low dosage (80 ppm) ameliorated lung pathology in ventilator-induced lung injury [7]. In a related model, Francis et al. demonstrated, that inhalation of 60 ppm was sufficient to exert a series of lung protective effects [24]. These data suggest that application of lower concentrations as we used can achieve lung protection. Although we did not detect any toxic side effects of H_2_S inhalation in our model (data not shown), future studies in this direction are needed to minimize H_2_S exposure.

### Hydrogen sulfide mediates lung protection by inhibition of the inflammatory response

**Figure 3:**
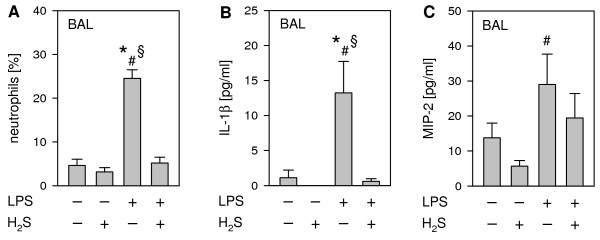
**Effect of LPS and hydrogen sulfide inhalation on lung inflammation.** As controls, mice received phosphate buffered saline (PBS, intranasally) and were kept in room air or in 80 ppm H_2_S for 6 h (plus 1 h pretreatment). LPS-treated mice (LPS treatment, i.n.) were either kept in room air or in 80 ppm H_2_S for 6 h (plus 1 h pretreatment). Bronchoalveolar lavage was performed in the right lung. The relative amount of neutrophils (**A**) was determined by cytospin analysis, and the amount of IL-1β (**B**) and MIP-2 (**C**) was determined by ELISA. Graphs represent means ± SEM, *n* = 8/group. ANOVA on Ranks (Dunn`s posthoc test), *P < 0.05 *vs*. PBS + air group; ^#^*P* < 0.05 *vs*. PBS + H_2_S group; ^§^*P* < 0.05 *vs*. LPS + H_2_S group.

We next investigated whether the observed H_2_S-mediated lung-protection was attributed to inhibition of LPS-induced inflammation. The development of lung damage in pulmonary sepsis is known to be critically dependent on the initiation of an inflammatory response, mainly characterized by neutrophil transmigration and activation and pro-inflammatory cytokine release [21,25]. Neutrophil sequestration into the bronchoalveolar space was markedly increased by LPS application as compared to the control (Figure 3A). H_2_S administration in LPS treated mice substantially reduced neutrophil numbers to control levels. Our data are in line with recent publications, where pulmonary neutrophil activity was found to be largely decreased in mice or rats, that had been protected from LPS-induced systemic inflammation both by inhaled H_2_S [23] as well as by application of the slowly releasing H_2_S donors S-diclofenac and GYY4137 [25,26]. The findings of these trials clearly support the notion that exogenous H_2_S can inhibit pro-inflammatory processes. In combination with neutrophil transmigration, the release of pro-inflammatory cytokines, *e.g.*, IL-1β and MIP-2, is known to aggravate lung injury [21,25]. In our study, quantitative analysis of IL-1β in the BAL revealed that it was nearly absent in both control groups (PBS + air and PBS + H_2_S), whereas LPS instillation alone led to a vast increase of IL-1β readings (Figure 3B). In sharp contrast, H_2_S inhalation reduced IL-1β to control levels. Likewise, LPS treatment increased MIP-2 protein that was partially prevented by H_2_S (Figure 3C). The attenuation of neutrophil transmigration and pro-inflammatory cytokine release by administration of H_2_S has also been shown in other models of ALI, *e.g.*, ventilator-induced lung injury [7], oleic acid-induced lung injury [9,10], caerulein-induced acute pancreatitis [27], or myocardial ischemia/reperfusion injury [28], strongly supporting our findings that gaseous H_2_S substantially inhibits pulmonary inflammation and thereby limits LPS-induced lung damage. Future studies using different ALI models might discover the regulatory role of H_2_S in each single aspect of human ALI / ARDS in order to define H_2_S` therapeutic potential.

**Figure 4:**
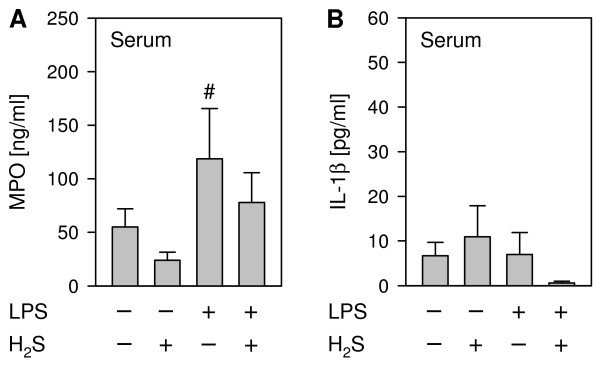
**Effect of LPS and hydrogen sulfide inhalation on systemic inflammation.** As controls, mice received phosphate buffered saline (PBS, intranasally) and were kept in room air or in 80 ppm H_2_S for 6 h (plus 1 h pretreatment). LPS-treated mice (LPS treatment, i.n.) were either kept in room air or in 80 ppm H_2_S for 6 h (plus 1 h pretreatment). Blood samples were withdrawn by intracardiac punctation. Myeloperoxidase (MPO, **A**) and IL-1β (**B**) contents were quantified by ELISA in serum. Graphs represent means ± SEM, *n* = 6-8/group. ANOVA on Ranks (Dunn`s posthoc test), ^#^*P* < 0.05 *vs*. PBS + H_2_S group.

We finally questioned whether intranasal application of LPS would also induce systemic inflammation and whether H_2_S would exert any systemic effects. As a marker for neutrophil activity we determined the concentration of the MPO glycoprotein in serum [23,26]. In the present study, LPS treatment clearly increased serum MPO release. H_2_S inhalation tended to reduce MPO levels, irrespective of the mode of additional PBS- or LPS-treatment (Figure 4A). Similar results were obtained by analyzing serum IL-1β levels (Figure 4B). Our findings compliment the results of two previous studies, where LPS-induced systemic inflammation (*e.g.*, plasma nitrite/nitrate levels, pro-inflammatory cytokine release) was also clearly prevented by H_2_S inhalation [23,29]. However, on the basis of our results, we cannot clearly decipher, whether i.n. LPS directly induced a systemic inflammation or whether the inflammatory response observed resulted from lung injury. Therefore two scenarios concerning the role of H_2_S appear possible: (1) H_2_S inhalation may directly inhibit lung and systemic inflammation, or (2) H_2_S inhalation may primarily inhibit lung inflammatory responses, secondary preventing a systemic inflammation. Either way, our data suggest that H_2_S inhalation has the potential to inhibit both local as well as systemic inflammatory responses to septic insults.

## Conclusions

In our model, inhalation of hydrogen sulfide substantially reduced LPS-induced acute lung injury. The observed protection appears to be mediated by the anti-inflammatory effects of H_2_S, *i.e.*, inhibition of neutrophil transmigration and pro-inflammatory cytokine release. Therefore, H_2_S application displays organ protective properties.

## Competing interests

The authors declare that they have no competing interests.

## Authors` contributions

SF conducted the study and wrote the manuscript; KKZ helped to conduct the study and analyzed the data; KMS helped to conduct the study; HE helped to analyze the data and critically revised the manuscript; RS helped to design and conduct the study and to analyze the data; HB helped to analyze the data and to write the manuscript; SGS helped to conduct the study; AH designed and conducted the study, analyzed the data and wrote the manuscript. All authors read and approved the final manuscript.

## References

[B1] Rubenfeld GD Caldwell E Peabody E Weaver J Martin DP Neff M Stern EJ Hudson LD Incidence and outcomes of acute lung injury N Engl J Med 2005 353 1685 1693 10.1056/NEJMoa050333 16236739

[B2] Tsushima K King LS Aggarwal NR De Gorordo A D'Alessio FR Kubo K Acute lung injury review Intern Med 2009 48 621 630 10.2169/internalmedicine.48.1741 19420806

[B3] Wheeler AP Bernard GR Acute lung injury and the acute respiratory distress syndrome: a clinical review Lancet 2007 369 1553 1564 10.1016/S0140-6736(07)60604-7 17482987

[B4] Martin GS Mannino DM Eaton S Moss M The epidemiology of sepsis in the United States from 1979 through 2000 N Engl J Med 2003 348 1546 1554 10.1056/NEJMoa022139 12700374

[B5] Matute-Bello G Frevert CW Martin TR Animal models of acute lung injury Am J Physiol Lung Cell Mol Physiol 2008 295 L379 L399 10.1152/ajplung.00010.2008 18621912 PMC2536793

[B6] Whiteman M Le Trionnaire S Chopra M Fox B Whatmore J Emerging role of hydrogen sulfide in health and disease: critical appraisal of biomarkers and pharmacological tools Clin Sci (Lond) 2011 121 459 488 10.1042/CS20110267 21843150

[B7] Faller S Ryter SW Choi AM Loop T Schmidt R Hoetzel A Inhaled hydrogen sulfide protects against ventilator-induced lung injury Anesthesiology 2010 113 104 115 10.1097/ALN.0b013e3181de7107 20574227

[B8] Biermann J Lagreze WA Schallner N Schwer CI Goebel U Inhalative preconditioning with hydrogen sulfide attenuated apoptosis after retinal ischemia/reperfusion injury Mol Vis 2011 17 1275 1286 21633713 PMC3103742

[B9] Li T Zhao B Wang C Wang H Liu Z Li W Jin H Tang C Du J Regulatory effects of hydrogen sulfide on IL-6, IL-8 and IL-10 levels in the plasma and pulmonary tissue of rats with acute lung injury Exp Biol Med (Maywood ) 2008 233 1081 1087 10.3181/0712-RM-354 18535161

[B10] Wang C Wang HY Liu ZW Fu Y Zhao B Effect of endogenous hydrogen sulfide on oxidative stress in oleic acid-induced acute lung injury in rats Chin Med J (Engl ) 2011 124 3476 3480 22340161

[B11] Faller S Foeckler M Strosing KM Spassov S Ryter SW Buerkle H Loop T Schmidt R Hoetzel A Kinetic effects of carbon monoxide inhalation on tissue protection in ventilator-induced lung injury Lab Invest 2012 92 7 999 1012 10.1038/labinvest.2012.55 22449795 PMC9812657

[B12] Ang SF Moochhala SM Bhatia M Hydrogen sulfide promotes transient receptor potential vanilloid 1-mediated neurogenic inflammation in polymicrobial sepsis Crit Care Med 2010 38 619 628 10.1097/CCM.0b013e3181c0df00 19851090

[B13] Ang SF Sio SW Moochhala SM MacAry PA Bhatia M Hydrogen sulfide upregulates cyclooxygenase-2 and prostaglandin E metabolite in sepsis-evoked acute lung injury via transient receptor potential vanilloid type 1 channel activation J Immunol 2011 187 4778 4787 10.4049/jimmunol.1101559 21957141

[B14] Ang SF Moochhala SM MacAry PA Bhatia M Hydrogen sulfide and neurogenic inflammation in polymicrobial sepsis: involvement of substance P and ERK-NF-kappaB signaling PLoS One 2011 6 e24535 10.1371/journal.pone.0024535 21931742 PMC3171449

[B15] Li L Bhatia M Zhu YZ Zhu YC Ramnath RD Wang ZJ Anuar FB Whiteman M Salto-Tellez M Moore PK Hydrogen sulfide is a novel mediator of lipopolysaccharide-induced inflammation in the mouse FASEB J 2005 19 1196 1198 15863703 10.1096/fj.04-3583fje

[B16] Zhang H Zhi L Moore PK Bhatia M Role of hydrogen sulfide in cecal ligation and puncture-induced sepsis in the mouse Am J Physiol Lung Cell Mol Physiol 2006 290 L1193 L1201 10.1152/ajplung.00489.2005 16428267

[B17] Zhang H Zhi L Moochhala S Moore PK Bhatia M Hydrogen sulfide acts as an inflammatory mediator in cecal ligation and puncture-induced sepsis in mice by upregulating the production of cytokines and chemokines via NF-kappaB Am J Physiol Lung Cell Mol Physiol 2007 292 L960 L971 17209138 10.1152/ajplung.00388.2006

[B18] Zhang H Hegde A Ng SW Adhikari S Moochhala SM Bhatia M Hydrogen sulfide up-regulates substance P in polymicrobial sepsis-associated lung injury J Immunol 2007 179 4153 4160 17785854 10.4049/jimmunol.179.6.4153

[B19] Zhang H Zhi L Moochhala SM Moore PK Bhatia M Endogenous hydrogen sulfide regulates leukocyte trafficking in cecal ligation and puncture-induced sepsis J Leukoc Biol 2007 82 894 905 10.1189/jlb.0407237 17599903

[B20] Zhang H Moochhala SM Bhatia M Endogenous hydrogen sulfide regulates inflammatory response by activating the ERK pathway in polymicrobial sepsis J Immunol 2008 181 4320 4331 18768890 10.4049/jimmunol.181.6.4320

[B21] Huang XL Zhou XH Zhou JL Ding CH Xian XH [Role of polymorphonuclear neutrophil in exogenous hydrogen sulfide attenuating endotoxin-induced acute lung injury.] Sheng Li Xue Bao 2009 61 356 360 19701587

[B22] Spiller F Orrico MI Nascimento DC Czaikoski PG Souto FO Alves-Filho JC Freitas A Carlos D Montenegro MF Neto AF Hydrogen sulfide improves neutrophil migration and survival in sepsis via K + ATP channel activation Am J Respir Crit Care Med 2010 182 360 368 10.1164/rccm.200907-1145OC 20339148

[B23] Tokuda K Kida K Marutani E Crimi E Bougaki M Khatri A Kimura H Ichinose F Inhaled hydrogen sulfide prevents endotoxin-induced systemic inflammation and improves survival by altering sulfide metabolism in mice Antioxid Redox Signal 2012 17 11 21 10.1089/ars.2011.4363 22221071 PMC3342565

[B24] Francis RC Vaporidi K Bloch KD Ichinose F Zapol WM Protective and Detrimental Effects of Sodium Sulfide and Hydrogen Sulfide in Murine Ventilator-induced Lung Injury Anesthesiology 2011 115 1012 1021 10.1097/ALN.0b013e31823306cf 21912243 PMC3752661

[B25] Li L Rossoni G Sparatore A Lee LC Del Soldato P Moore PK Anti-inflammatory and gastrointestinal effects of a novel diclofenac derivative Free Radic Biol Med 2007 42 706 719 10.1016/j.freeradbiomed.2006.12.011 17291994

[B26] Li L Salto-Tellez M Tan CH Whiteman M Moore PK GYY4137, a novel hydrogen sulfide-releasing molecule, protects against endotoxic shock in the rat Free Radic Biol Med 2009 47 103 113 10.1016/j.freeradbiomed.2009.04.014 19375498

[B27] Sidhapuriwala JN Ng SW Bhatia M Effects of hydrogen sulfide on inflammation in caerulein-induced acute pancreatitis J Inflamm (Lond) 2009 6 35 10.1186/1476-9255-6-35 20040116 PMC2804662

[B28] Sodha NR Clements RT Feng J Liu Y Bianchi C Horvath EM Szabo C Stahl GL Sellke FW Hydrogen sulfide therapy attenuates the inflammatory response in a porcine model of myocardial ischemia/reperfusion injury J Thorac Cardiovasc Surg 2009 138 977 984 10.1016/j.jtcvs.2008.08.074 19660398 PMC2758694

[B29] Wagner F Wagner K Weber S Stahl B Knoferl MW Huber-Lang M Seitz DH Asfar P Calzia E Senftleben U Inflammatory effects of hypothermia and inhaled H2S during resuscitated, hyperdynamic murine septic shock Shock 2011 35 396 402 10.1097/SHK.0b013e3181ffff0e 20938376

